# Predicting cancer drug TARGETS - TreAtment Response Generalized Elastic-neT Signatures

**DOI:** 10.1038/s41525-021-00239-z

**Published:** 2021-09-21

**Authors:** Nicholas R. Rydzewski, Erik Peterson, Joshua M. Lang, Menggang Yu, S. Laura Chang, Martin Sjöström, Hamza Bakhtiar, Gefei Song, Kyle T. Helzer, Matthew L. Bootsma, William S. Chen, Raunak M. Shrestha, Meng Zhang, David A. Quigley, Rahul Aggarwal, Eric J. Small, Daniel R. Wahl, Felix Y. Feng, Shuang G. Zhao

**Affiliations:** 1 Department of Human Oncology, University of Wisconsin, Madison, WI USA; 2 Department of Radiation Oncology, University of Michigan, Ann Arbor, MI USA; 3 Carbone Cancer Center, University of Wisconsin, Madison, WI USA; 4 Department of Medicine, University of Wisconsin, Madison, WI USA; 5 Department of Biostatistics and Medical Informatics, University of Wisconsin, Madison, WI USA; 6 Department of Radiation Oncology, UCSF, San Francisco, CA USA; 7 Helen Diller Family Comprehensive Cancer Center, UCSF, San Francisco, CA USA; 8 Department of Epidemiology and Biostatistics, UCSF, San Francisco, CA USA; 9 Division of Hematology and Oncology, Department of Medicine, UCSF, San Francisco, CA USA; 10 Department of Urology, UCSF, San Francisco, CA USA; 11 William S. Middleton Memorial Veterans Hospital, Madison, WI USA

**Keywords:** Bioinformatics, Targeted therapies, Predictive markers, Prognostic markers, Molecular medicine

## Abstract

We are now in an era of molecular medicine, where specific DNA alterations can be used to identify patients who will respond to specific drugs. However, there are only a handful of clinically used predictive biomarkers in oncology. Herein, we describe an approach utilizing in vitro DNA and RNA sequencing and drug response data to create TreAtment Response Generalized Elastic-neT Signatures (TARGETS). We trained TARGETS drug response models using Elastic-Net regression in the publicly available Genomics of Drug Sensitivity in Cancer (GDSC) database. Models were then validated on additional in-vitro data from the Cancer Cell Line Encyclopedia (CCLE), and on clinical samples from The Cancer Genome Atlas (TCGA) and Stand Up to Cancer/Prostate Cancer Foundation West Coast Prostate Cancer Dream Team (WCDT). First, we demonstrated that all TARGETS models successfully predicted treatment response in the separate in-vitro CCLE treatment response dataset. Next, we evaluated all FDA-approved biomarker-based cancer drug indications in TCGA and demonstrated that TARGETS predictions were concordant with established clinical indications. Finally, we performed independent clinical validation in the WCDT and found that the TARGETS AR signaling inhibitors (ARSI) signature successfully predicted clinical treatment response in metastatic castration-resistant prostate cancer with a statistically significant interaction between the TARGETS score and PSA response (*p* = 0.0252). TARGETS represents a pan-cancer, platform-independent approach to predict response to oncologic therapies and could be used as a tool to better select patients for existing therapies as well as identify new indications for testing in prospective clinical trials.

## Introduction

Treatment decisions for cancer patients have historically depended on the tumor location and histologic appearance. However, response is often heterogeneous within the same tumor type^1^. Molecular diversity is fundamental to a cancer’s ability to evade endogenous and exogenous tumor control strategies, and there is a great need to incorporate an understanding of this diversity into the management of all cancer patients. Advances in next-generation sequencing have ushered in a personalized treatment approach that can improve tumor control and decrease side effects compared to the traditional one-size-fits-all approach.

Multiple anti-neoplastic therapies have now been paired with predictive biomarkers for making treatment decisions. This approach has been particularly successful with targeted drug therapies. The first successful examples include Imatinib for chronic myelogenous leukemia patients with the BCR-ABL fusion^2^ and Trastuzumab for HER2-positive breast cancer patients^3^. Since the approval of these agents 20 years ago, the FDA has approved dozens of different targeted therapies, with the number increasing rapidly every year. However, even among these targeted therapies and among patients who have a mutation known to confer increased sensitivity to the therapy, treatment outcomes can still be heterogeneous. For example, even among non-small cell lung cancer (NSCLC) patients with classic EGFR mutations, where exon 19 deletions and L858R exon 21 point mutations account for 90% of EGFR mutations, response rates have ranged from 58 to 85% in phase IIb/III clinical trials evaluating anti-EGFR tyrosine kinase inhibitors (e.g., Erlotinib, Gefitinib, Afatinib, Osimertinib)^4–11^.

A contributing factor to variability in treatment response is the complex and often compound nature of cancer gene alterations. Multiple mutations and gene expression differences likely modulate response, but many of the relevant changes are challenging to identify. We hypothesized that next-generation DNA and RNA sequencing techniques paired with modern computational modeling could identify gene signatures that would better capture this heterogeneity. Rather than relying on the presence or absence of a single genetic variant, we instead model treatment predictions based on a broad spectrum of genomic variant and expression data. To do this, we have leveraged an existing large-scale in-vitro database to train TreAtment Response Generalized Elastic-neT Signatures (TARGETS). We then validated these results on three independent cohorts. First, we showed concordant drug-response predictions in an external in-vitro database. Second, we demonstrated that our predictions were concordant with known FDA biomarkers-drug indications in a large cohort of sequenced tumors. Third, we validated TARGETS as a predictive biomarker of androgen receptor signaling inhibitor (ARSI) response in a unique dataset of metastatic prostate cancer patients. Finally, we evaluated the utility of TARGETS as a tool for targeted hypothesis generation in identifying new drug indications. This pan-cancer, platform-independent approach can be used to better identify responders vs. non-responders and could potentially identify new patient populations which would benefit from specific treatments.

## Results

### Training models on the GDSC database

**Fig. 1:**
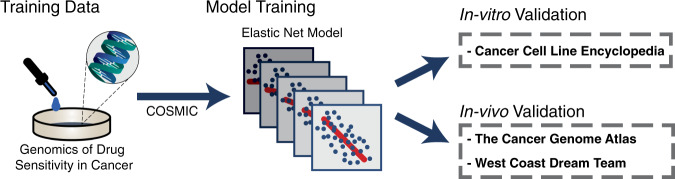
TARGETS Workflow. Flowchart depicting TARGETS model training on DNA and RNA sequencing data from the Genomics of Drug Sensitivity in Cancer (GDSC) database to predict drug response. Model training was performed utilizing Elastic Net regression. The drug models were locked and subsequently validated on three independent cohorts: the Cancer Cell Line Encyclopedia (CCLE), The Cancer Genome Atlas (TCGA) pan-cancer database, and the West Coast Dream Team (WCDT) metastatic prostate cancer database.

Our training cohort was the publicly available Genomics of Drug Sensitivity in Cancer (GDSC) database^12–14^ (Fig. 1). To reduce the noise in the data, we included only genes identified by the COSMIC Cancer Gene Census^15^. This critical step allowed us to leverage the extensive knowledge on cancer genomics to improve the signal-to-noise ratio and prediction accuracy. Elastic-Net regression models were then trained using the RNA expression and DNA mutation data on only the COSMIC genes for all treatments in the GDSC. The TARGETS models were locked and used for all subsequent predictions without modification.

### Concordance with CCLE drug sensitivity

We next examined if the TARGETS predictions could successfully predict cell line drug response in an independent dataset from the Cancer Cell Line Encyclopedia (CCLE)^16^. Eighteen drugs were present in both CCLE and GDSC, allowing us to independently validate the performance of those 18 TARGETS models in CCLE. We compared the TARGETS predictions with the drug sensitivities in CCLE and found that 18 out of 18 were significantly correlated after adjusting for multiple testing (FDRs <0.05, Table 1). Validation of all models in an independent cell line drug response cohort provides additional experimental evidence supporting the TARGETS approach.

**Table 1:** TARGETS predictions correlate with CCLE drug sensitivity.

Drug	Corr. coef.	*P*-value	FDR
Nilotinib	0.6 [0.66, 0.53]	5.95E−38	2.14E−37
Tanespimycin	0.25 [0.33, 0.16]	8.95E−08	1.07E−07
PHA-665752	0.24 [0.33, 0.15]	1.45E−07	1.63E−07
Lapatinib	0.49 [0.56, 0.42]	4.06E−29	9.13E−29
Nutlin-3a (-)	0.26 [0.35, 0.18]	1.11E−08	1.43E−08
Saracatinib	0.22 [0.31, 0.13]	1.38E−06	1.46E−06
Crizotinib	0.45 [0.52, 0.37]	6.06E−24	1.09E−23
Panobinostat	0.61 [0.67, 0.55]	1.83E−48	3.29E−47
Sorafenib	0.42 [0.49, 0.34]	1.45E−20	2.18E−20
Irinotecan	0.65 [0.71, 0.57]	1.11E−35	3.33E−35
Topotecan	0.58 [0.64, 0.52]	3.97E−43	3.57E−42
PD0325901	0.57 [0.63, 0.51]	1.27E−41	7.63E−41
Palbociclib	0.23 [0.32, 0.14]	3.45E−06	3.45E−06
Paclitaxel	0.51 [0.57, 0.44]	2.97E−31	7.64E−31
Selumetinib	0.48 [0.55, 0.4]	1.51E−27	3.03E−27
PLX-4720	0.57 [0.63, 0.51]	7.66E−41	3.45E−40
NVP-TAE684	0.34 [0.42, 0.26]	6.76E−14	9.36E−14
Erlotinib	0.45 [0.52, 0.37]	1.13E−23	1.84E−23

Pearson’s correlation between TARGETS predictions in CCLE and drug sensitivity as measured by the negative AUC.

*FDR* Benjamini Hochberg corrected false discovery rate.

### Concordance with Known biomarker-drug combinations in the TCGA

**Fig. 2:**
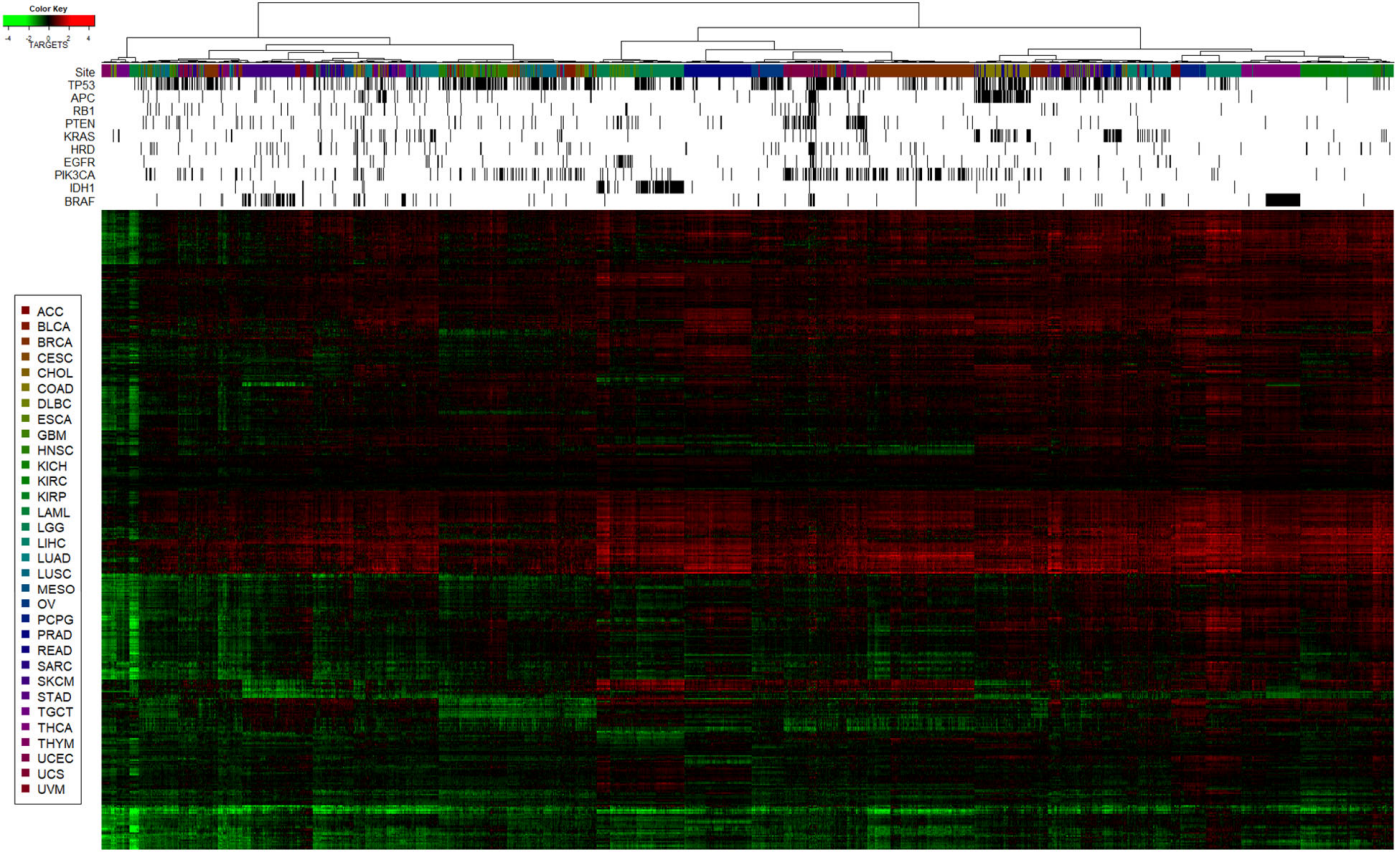
TARGETS Scores in TCGA patients. TARGETS predictions for all drugs (rows) across all samples in the TCGA (columns) are shown in heatmap form. Hierarchical clustering was performed on both rows and columns. A lower TARGETS score indicates predicted sensitivity. Cancer site and type is identified using the TCGA standardized study abbreviations.

**Fig. 3:**
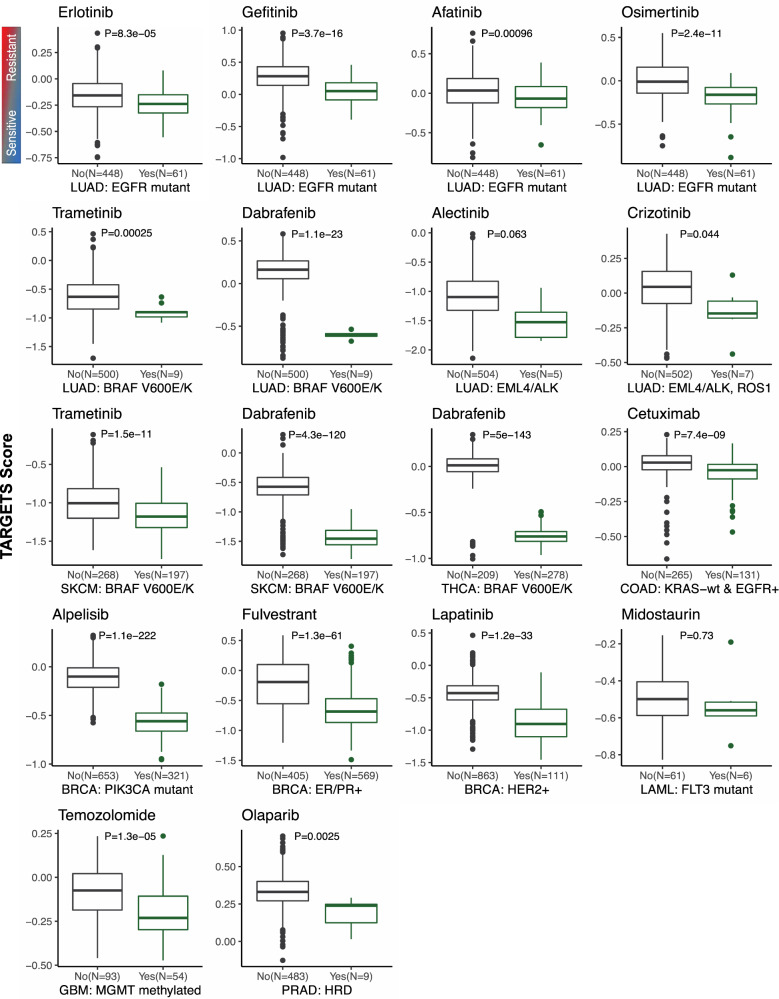
TARGETS concordance with FDA-approved and clinically used biomarker indications. Boxplots comparing predicted TARGETS drug sensitivity among patients with or without a specific biomarker. Distributions were compared using an unpaired two-sample *t*-test, with *p*-value reported. A lower TARGETS score indicates increased predicted sensitivity. Cancer site and type for each plot is identified using the TCGA standardized study abbreviations. Boxplot center line = median; box limits = upper and lower quartiles; whiskers = 1.5× interquartile range.

Data on 9430 patients from 32 cancer types from The Cancer Genome Atlas (TCGA)^17^ was used to compare TARGETS with known biomarker-drug combinations. The distribution of predicted sensitivities varies widely across tumors and drugs. When we plotted the TARGETS predictions for all drugs across all tumor types, we observed that samples with the same tumor types tended to cluster together, as well as certain DNA alterations which tend to be highly enriched in certain tumor types (Fig. 2). This is consistent with the evidence that many anti-cancer drugs tend to work better in specific tumor types, an assumption underlying current clinical practice. However, there is also a minority of samples that appear to be dissimilar to their tissue-of-origin and cluster better with other tumor types, highlighting the limitation of tumor-type-driven treatment decisions and the potential benefit of a molecularly driven approach. Predictions of drug sensitivity using TARGETS were made for all drugs and samples. We next tested our TARGETS predictions against all FDA-approved somatic biomarker indications (Supplementary Data 1). For all biomarker-drug combinations tested, differences in drug sensitivity as predicted by TARGETS were in line with what was expected based on the indication (Fig. 3). EGFR mutated lung adenocarcinomas were predicted to be more sensitive to Erlotinib, Gefitinib, Afatinib and Osimertinib (all with *p* < 0.0001). BRAF V600E/K mutated lung adenocarcinoma and cutaneous melanoma both were predicted to be more sensitive to Trametinib and Dabrafenib (all with *p* < 0.001). BRAF V600E/K mutant thyroid cancer was also predicted to be more sensitive to Dabrafenib (*p* < 0.0001). EML4/ALK fusion-positive lung adenocarcinoma was predicted to be borderline more sensitive to Alectinib (*p* = 0.0632) and EML4/ALK or ROS1 fusion-positive lung adenocarcinoma was predicted to be more sensitive to Crizotinib (*p* = 0.044). KRAS wild-type colon cancer with EGFR expression greater than the median was predicted to be more resistant to Cetuximab (*p* < 0.0001). PIK3CA mutated breast tumors were predicted to be more sensitive to Alpelisib. ER/PR positive breast cancer by histologic assessment was predicted to be more sensitive to Fulvestrant (an ER degrader, *p* < 0.0001) and HER2 positive breast cancer by histologic assessment was predicted to be more sensitive to Lapatinib (*p* < 0.0001). Midostaurin was not predicted to be significantly more sensitive in FLT3 mutant AML. However, the complete response rate even in FLT3 wild-type AML treated with Midostaurin can be up to 74%^18,19^. In addition to these FDA-approved indications, we tested other clinically used biomarker-drug combinations. In GBM, the benefit of Temozolomide is more pronounced in MGMT promoter methylated tumors^20–23^, and we found MGMT-methylated glioblastoma was predicted to be more sensitive to Temozolomide (*p* < 0.0001). PARP inhibitors, such as Olaparib, are now indicated for both HRD and non-HRD ovarian cancers, and we also did not find a significant difference in sensitivity to Olaparib between HRD and non-HRD ovarian cancers^24^. However, in prostate cancer, HRD tumors were predicted to be more sensitive to Olaparib (*p* = 0.0025), consistent with recent data from the phase III PROfound trial^25^. These data therefore provide independent evidence that TARGETS predictions are concordant with FDA-approved biomarker indications.

### Predicting ARSI response in metastatic prostate cancer

**Fig. 4:**
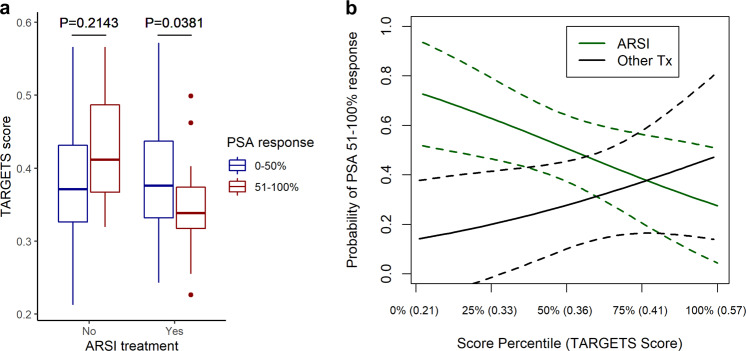
Predicting response to ARSIs in mCRPC. **a** Patients receiving ARSIs who had a 51–100% response in PSA were predicted to be more sensitive by TARGETS than those with a 0–50% response. There was no difference in predicted sensitivity in those not treated. **b** Interaction plot showing the probability of PSA 51–100% response as a function of TARGETS score in the patients treated with an ARSI vs. other treatments. A lower TARGETS score indicates predicted sensitivity. Boxplot center line = median; box limits = upper and lower quartiles; whiskers = 1.5× interquartile range.

Metastatic castration-resistant prostate cancer (mCRPC) is a common lethal cancer type not represented in the TCGA, and is commonly treated with ARSIs such as Enzalutamide or Abiraterone. This cancer type represents an opportunity to clinically validate our approach in an independent patient cohort. We utilized metastatic biopsy RNA and DNA sequencing data as well as ARSI response data on 100 patients from the Stand Up to Cancer/Prostate Cancer Foundation West Coast Prostate Cancer Dream Team (WCDT) cohort^26^ to evaluate whether TARGETS could predict which patients may benefit from ARSI therapy. 50% PSA response is a common cutoff used in randomized trials in metastatic prostate cancer^27–31^, and we used this as our primary clinical endpoint. We found that among patients receiving ARSIs as the next-line therapy after their biopsy, responders (defined as those who had 51–100% PSA response) were predicted to be more sensitive to ARSIs compared to the non-responders (0–50% PSA response) (Fig. 4a; *p* = 0.0381). There was no difference in the predicted sensitivity to ARSIs of responders and non-responders who received other drugs (*p* = 0.2143), providing a control that shows the model is specific in identifying patients who will respond to ARSIs rather than just identifying those who will have a good response to treatment in general. In a logistic regression model predicting PSA response, the interaction between ARSI treatment and TARGETS score was statistically significant (*p* = 0.0252; Fig. 4b) indicating that TARGETS is a *bona fide* predictive biomarker for response to ARSIs^32–36^.

### Exploratory identification of potential therapeutic strategies with TARGETS

**Fig. 5:**
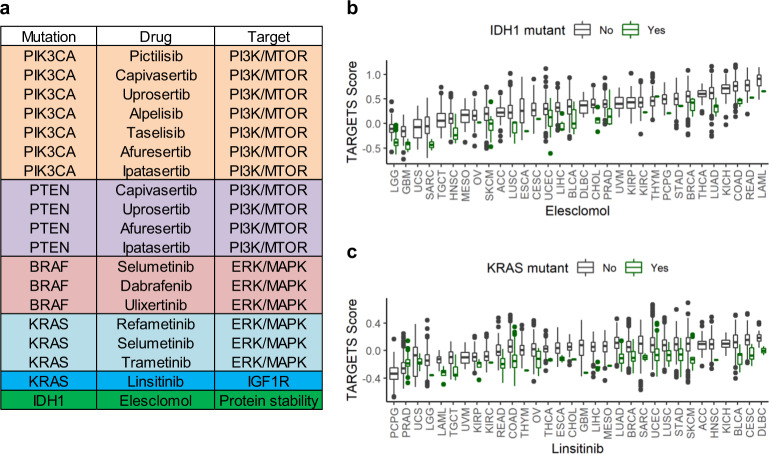
Novel mutations predicted to confer drug sensitivity. **a** List of top 1% mutations predicted to confer sensitivity to a specific agent across tumor types. Linear model *p*-values all <0.0001. The rank is based on the normalized weighting of the model with each model. **b** Differences in TARGETS scores across 32 cancer types for Linsitinib. **c** Differences in TARGETS scores across 32 cancer types for Elesclomol. A lower TARGETS score indicates predicted sensitivity. Boxplot center line = median; box limits = upper and lower quartiles; whiskers = 1.5× interquartile range.

While mutations may occur randomly, those that provide a growth advantage are selected for in cancer. Frequent mutation of a gene may signal a tumor’s dependence on that gene or pathway and therefore represents a potential therapeutic target. We hypothesized that examining specific mutations associated with TARGETS in clinical samples could identify known and novel therapeutic strategies. To this end, we identified the mutations most strongly correlated with TARGETS predictions in TCGA. The top 1% of putative mutation–drug sensitivity combinations are shown in Fig. 5a. Out of these 19 pairs, 17 were associations that would be reasonably expected given their mechanism of action (e.g., PIK3CA/PTEN mutations and PI3K/MTOR inhibitors, BRAF/KRAS mutations, and ERK/MAPK inhibitors). Overall, tumors with PIK3CA and PTEN mutations were predicted to be more sensitive to drugs that target the PI3K/MTOR pathway which is downstream of those genes. Tumors with KRAS and BRAF mutations were predicted to be more sensitive to drugs that target the ERK/MAPK pathway which is downstream of RAS/RAF signaling. In addition, Linsitinib, an IGF1R inhibitor, was predicted to be more effective in KRAS mutant tumors (Fig. 5b), consistent with experimental data in NSCLC^37^. The final drug on the list, Elesclomol, was predicted to be more effective in IDH1 mutant tumors, especially gliomas (Fig. 5c), an association not previously reported in the literature. There were no IDH1 mutant LGG or GBM cell lines included in the GDSC, but TARGETS was nonetheless able to identify improved predicted Temozolomide response in MGMT methylated GBM patients (Fig. 3). These predictions represent hypothesis-generating extrapolations that go beyond the original training data, which can be used to identify potential novel therapeutic strategies.

## Discussion

Personalized genomic medicine has changed the paradigm of cancer treatment. Next-generation genomic sequencing has shifted treatment decisions from using radiologic and histologic data alone, to an approach that incorporates individualized molecular features. In this study, we set out to develop TARGETS, a pan-cancer, platform-independent model for predicting sensitivity to therapy based on RNA expression and DNA mutation profiles. TARGETS was then validated across three datasets: the in-vitro CCLE and in vivo TCGA and WCDT datasets. Our predicted results were concordant for all 18 drugs that were common between the CCLE and GDSC, and TARGETS had consistent predictions with known biomarker-drug indications across the TCGA. Furthermore, we independently validated TARGETS as a predictive biomarker for ARSI response in mCRPC in the WCDT cohort. Finally, we evaluated TARGETS use as a tool for hypothesis generation in identifying new drug indications.

Many attempts have been made to develop in vitro pharmacogenomic response signatures based on the publicly available GDSC, CCLE, and TCGA datasets^14,16,38–47^. TARGETS demonstrates a stronger level of concordance across all known biomarker-drug indications in clinical samples than has been described in previously published studies^48^. A few studies have also trained RNA-based signatures that were prognostic in clinical cohorts treated with specific agents^49–51^. However, these studies have not necessarily identified predictive biomarkers, which are biomarkers that predict response only to a particular treatment, thus requiring validation data that includes un-treated patients^32–36^. This distinction is particularly important with regards to non-targeted therapies, such as traditional cytotoxic chemotherapies, which have been the focus of most of these prior studies. When no un-treated patient data exists, a signature for “response” may simply be measuring the overall aggressiveness of a tumor (e.g., prognosis), instead of providing truly predictive information specific to that agent. Statistical interaction testing, as we demonstrate, is required to identify truly predictive biomarkers^32–36^.

The primary challenge in assessing the performance of TARGETS is locating suitable clinical validation datasets with both multi-omics and treatment response data. There are in vitro pharmacogenomic databases such as the CCLE in which we were able to perform validation. The CCLE is similar to the GDSC, including many shared cell lines as both were designed to be comprehensive catalogues of cancer cell lines. However, the two cohorts were distinct efforts in time and space, and there were significant differences in culture conditions, gene expression profiling, drug screen procedures, and many other major and minor factors, to the extent that significant discordance between the two datasets has been reported^52,53^. The validation of 100% of TARGETS predictions in CCLE despite these differences provides strong supporting evidence for the approach. Ideally, clinical validation would be performed for every drug in every disease site. However, there is a lack of clinical cohorts with both DNA and RNA sequencing and detailed response data from both treated and untreated patients. Datasets such as the TCGA have the former but not the latter. Furthermore, systemic therapies are primarily used in the later stages of the disease, but obtaining invasive metastatic biopsies for molecular profiling is not routine. The WCDT is a unique cohort with both comprehensive molecular profiling and ARSI drug response data making it the ideal clinical dataset in which to validate TARGETS. The rarity of such clinical datasets highlights the need for DNA and RNA profiling in larger prospective studies with detailed treatment and outcomes data.

We believe the model development strategy presented herein has yielded improved generalizability and interpretability. First, our approach is unique in that we use only genes known to be strongly associated with cancer from the literature^15^. While it initially seems counter-intuitive that removing information from the vast majority of genes would be beneficial, a genome-wide approach suffers from a great deal of noise. Not only are many genes not important to treatment response or resistance, but cell lines in particular acquire many passenger mutations over time. Therefore, by focusing on a small set of cancer-associated genes, changes in gene expression or the presence of mutations are more likely to be driving a biological function. Second, integration of both DNA and RNA information into our models can provide information on tumors driven by specific gene expression patterns (e.g., receptors in breast cancer) as well as specific DNA alterations (e.g., EGFR mutations in lung cancer)^46,54^. Finally, we chose to utilize Elastic-Net regression^55^, because this regularized approach is less prone to over-fitting^56^ and thus would better handle the biological and technical differences between the in-vitro training data and the clinical datasets.

TARGETS may also be able to identify new therapeutic strategies. Interestingly, our results show that IDH1 mutations are the second most highly weighted feature in the model for Elesclomol, and that they are highly associated with predicted Elesclomol sensitivity. Elesclomol is a copper chelator that has been found to interact with the electron transport chain in mitochondria to generate high levels of reactive oxygen species (ROS)^57^. IDH1 is well known for its role in the NADPH-dependent catalyzation of isocitrate to a-ketoglutarate (aKG), with IDH1 mutations leading to NADPH-dependent reduction of aKG to D-2-hydroxyglutarate (D2HG)^58^. While D2HG has many downstream effects that contribute to tumorigenesis in IDH mutant tumors^59^, this increased utilization of NADPH impacts the cell’s ability to form a sufficient response to increased production of ROS. This mechanism could in part explain why IDH1-mutant glioma patients have better prognosis^60^ and would mechanistically support our prediction of increased sensitivity to Elesclomol in IDH1-mutant tumors. To our knowledge, this association has not been previously documented in the literature and thus warrants further investigation to evaluate its use in IDH1-mutant tumors, particularly gliomas, which were predicted to have the greatest sensitivity to this agent with or without IDH1 mutation.

In conclusion, our study describes a pan-cancer, multi-omics approach for the identification of predictive biomarkers across tumor types. Many drugs demonstrate some efficacy in a minority of patients but lack sufficient clinical benefit in unselected populations to warrant FDA approval or clinical use. To date, we lack a unified global approach for identifying the patients most likely to benefit from specific therapies. TARGETS is platform-independent, and thus can be applied to a wide range of current and future datasets. RNA-seq should be normalized as described, and any DNA variant-calling pipeline can be used. There will of course be technical variation across different datasets. However, elastic-net regression is particularly well suited to handle some degree of noise, and our validation is on a variety of different platforms. TARGETS could be used in future clinical trials to select only patients most likely to benefit from the trial agent for inclusion, thus maximizing the chances of success.

## Methods

### Literature review of FDA approved somatic biomarker indications in cancer

To establish a comprehensive list of all clinically approved biomarker-drug combinations to analyze in this study, we obtained a list of United States Food and Drug Administration (FDA) pharmacogenomic indications (www.fda.gov/drugs/science-and-research-drugs/table-pharmacogenomic-biomarkers-drug-labeling, version dated 5 February 2020; Supplementary Data 1). In addition to the biomarker-drug combinations in the FDA list, we also examined clinically utilized MGMT promoter methylation with Temozolomide in glioblastoma^20–23^ and homologous recombination deficiency with Olaparib in prostate cancer^25^. While PARP inhibitors such as Olaparib are indicated for both HRD and non-HRD ovarian cancers^24^ and are also indicated for germline BRCA1/2 mutant breast cancer, germline variants are restricted data in the TCGA, and our focus was on somatic variants, so these germline indications were not assessed. EML4-ALK and ROS1 fusions were called using the Jackson Laboratory Tumor Fusion Gene Data Portal (www.tumorfusions.org)^61^. As fusion partners for ROS1 are less well defined, only ROS1 fusions confirmed by WGS were included. ER, PR, HER2 positivity, MGMT promoter methylation, and FLT3 mutation were defined by the TCGA phenotypic data. All other mutations were defined by the sequencing data. EGFR staining was not available, and so EGFR positivity was defined as greater than median EGFR expression, based on literature supporting a range of EGFR positivity of 25–82% in colorectal cancer^62^.

### Training in GDSC

Processed mutation calls and RNA-seq FPKM gene expression data on cancer cell lines publicly available through the GDSC were downloaded from the GDSC website (www.cancerrxgene.org)^12^. Mutations were coded as “present” only if they affected the protein-coding region of a gene (i.e., excluding silent, intronic, and inter-genic mutations), otherwise, they were coded as “not present”. Gene expression was Log_2_ transformed, scaled to the median of the cohort, and treated as a continuous variable. We filtered variant and expression data to focus on the 702 COSMIC cancer genes present on all platforms in the training and validation cohorts^15^. The GDSC database contains IC_50_ information for 449 drugs across 982 cell lines and DNA and RNA sequencing data for these cell lines. To develop a model for each drug in the database, we used Elastic-Net regression, a regularized regression method that is a linear combination of the LASSO and Ridge methods. The Elastic-Net regression model is a penalized approach that produces biased coefficient estimates with a resulting decrease in variance, which can lead to an improvement in predictions compared to what can be achieved with a non-penalized regression model. This method also allows for feature selection, with coefficients of non-predictive features falling to zero or near-zero. To determine the optimized trade-off in bias and variance, cross validation is utilized to tune the two hyper-parameters of this model: the strength of the penalization (λ) and the proportion of LASSO versus Ridge penalty (α). An Elastic-Net model^55^ was trained for all drugs in GDSC using the R caret wrapper for the GLMNET package, using the default parameters. Values for α and λ were selected using 10-fold cross validation. The reported Z-score of the half-maximal inhibitory concentration (IC_50_)^12^ of each drug experiment was used as the measure of response in our model. The final output model from the Elastic-Net training procedure is in the form of a standard linear model, and the intercept and coefficients of all models described below can be found in Supplementary Data 2. The predictions from these models represent the TARGETS scores. Of note, immunotherapies were not tested in the GDSC and are not represented in TARGETS because these depend on the interaction between the tumor and host immune system, which was not modeled in the GDSC cell line experiments.

### In vitro validation

Independent validation of cell line drug response predictions was performed in the CCLE dataset^16^. RNA and DNA sequencing data were downloaded from the CCLE website (portals.broadinstitute.org/ccle). Gene expression and mutation data were normalized and represented the same way as the GDSC, detailed above. Predictions were made using the locked models previously trained in GDSC. Eighteen previously trained drug-models from the GDSC had matching drug response data available in the CCLE. In the CCLE dataset, 55% of all the IC_50_ values were 8 μM (the maximal tested concentration). Thus, we utilized the AUC instead, which provides drug response information even if the IC_50_ was not reached. Since higher AUC is associated with lower IC_50_, we then compared the negative AUC determined from CCLE samples and compared to the GDSC predicted IC_50_ to determine the correlation between our two predictions. A Pearson’s correlation coefficient was determined for all 18 comparisons and the Benjamini Hochberg False Discovery Rate (FDR) was reported for each comparison to control for multiple testing.

### In vivo validation

TARGETS performance was evaluated in two clinical datasets: TCGA and the Stand Up to Cancer/Prostate Cancer Foundation WCDT. The TCGA processed sequencing and clinical data were downloaded using the UCSC Xena browser (xena.ucsc.edu)^63^. The WCDT dataset contains 100 patients with mCRPC with both DNA and RNA sequencing^26^, with Whole Genome Sequencing and RNA-seq data available at dbGAP (phs001648.v2.p1). We paired these data with previously unreported treatment response data to validate the ability of TARGETS to predict treatment response in this unique clinical cohort. Gene expression and mutation data were normalized and represented in the same manner as for both in-vitro datasets. Predictions were made with the GDSC-trained and locked models without modification. Comparisons of predicted Z-score IC_50_ between groups were performed using a *T*-test. Of note, the ARSI model as derived in GDSC was based on Bicalutamide, the only ARSI included in the training dataset. The ARSIs used in the WCDT were Enzalutamide and Abiraterone.

### Identifying novel biomarker-drug pairs

We utilized the TARGETS predictions detailed above to globally identify mutations associated with predicted drug sensitivity in TCGA. A linear model was used for this step, and tumor site was also included to identify pan-cancer biomarker-drug pairs. This approach identified mutations that were associated with drug sensitivity, independent of the disease site. Only named drugs further along in the regulatory process^64^ and mutations with a >5% frequency across all cancers were included. The *t*-statistic of the mutation in the linear model was used to rank the mutation-drug pairs, and the top 1% were selected for further investigation.

### Ethics statement

The GDSC, CCLE, and TCGA data utilized in this study are all available publicly and thus no institutional review was required for data acquisition. The WCDT was a multi-institutional prospective Institutional Review Board (IRB) approved study (NCT02432001), including a tissue acquisition and molecular profiling protocol, with all study participants providing written informed consent to participate^26^.

### Reporting summary

Further information on research design is available in the Nature Research Reporting Summary linked to this article.

## Supplementary information

Supplementary Data 1, 2

Reporting Summary

## Data Availability

The data that support the findings of this study are available through the following locations. The Genomics of Drug Sensitivity in Cancer (GDSC) data were downloaded from the GDSC website (www.cancerrxgene.org). The Cancer Cell Line Encyclopedia (CCLE) dataset were downloaded from the CCLE website (portals.broadinstitute.org/ccle). The TCGA processed sequencing and clinical data were downloaded using the UCSC Xena browser (xena.ucsc.edu). The WCDT dataset with Whole Genome Sequencing and RNA-seq data is available at dbGAP (phs001648.v2.p1). Additional clinical data from the WCDT will be made upon request.

## Code availability

The TARGETS models are represented by linear models, and the co-efficients are available in Supplementary Data 2 which allow for generation of the TARGETS scores. All analysis was performed using R 4.0.3. Models were generated using R “caret” package with the following parameters: method = glmnet, trControl = trainControl(method = “repeatedCV”, number = 10, repeats = 10).
